# Diagnostic Delay in Patients with Primary Sjögren’s Syndrome: A Population-Based Cohort Study in Taiwan

**DOI:** 10.3390/healthcare9030363

**Published:** 2021-03-23

**Authors:** Yu-Tung Huang, Tsung-Hsueh Lu, Pi-Ling Chou, Meng-Yu Weng

**Affiliations:** 1 Center for Big Data Analytics and Statistics, Chang Gung Memorial Hospital, Linkou, Taoyuan 333, Taiwan; anton.huang@gmail.com; 2 Institute of Public Health, College of Medicine, National Cheng Kung University, Tainan 701, Taiwan; robertlu@mail.ncku.edu.tw; 3 School of Nursing, College of Nursing, Kaohsiung Medical University, Kaohsiung 807, Taiwan; ling0319@kmu.edu.tw; 4 Department of Medical Research, Kaohsiung Medical University Hospital, Kaohsiung 807, Taiwan; 5 Section of Allergy, Immunology & Rheumatology, Department of Internal Medicine, National Cheng-Kung University Hospital, College of Medicine, National Cheng Kung University, Tainan 704, Taiwan

**Keywords:** primary Sjogren’s syndrome, diagnostic delay, population-based study

## Abstract

The diagnosis of primary Sjögren’s syndrome (pSS) can be challenging because the cardinal sicca syndromes may be subjective and subclinical. Diagnostic delay is common among patients with pSS. The aim of this study was to assess the time of lag between the onset of sicca symptoms and a subsequent diagnosis of pSS. We used population-based data from Taiwan’s National Health Insurance (NHI) claims directory spanning up to 6 years between 2006 and 2011. All NHI-covered patients receiving a first-time approved catastrophic illness certificate (CIC) for pSS in 2011 were included; their sicca symptoms and utilization of medical resources were then traced retrospectively over five years to 2006. The time of lag was identified by observing the onset of sicca symptoms, a diagnosis of Sjögren’s syndrome, and the related claim for CIC. A total of 1970 pSS patients were included in this study. The median time of lag between the onset of sicca symptoms and pSS diagnosis was 115 weeks (interquartile range [IQR] 27–205), and between pSS diagnosis and approval of CIC, was 6 (IQR 2–37) weeks. During the time of lag between sicca symptoms, diagnosis, and approval of a CIC for pSS, the median numbers of outpatient visits were 3 (IQR 1–8) and 3 (IQR 2–7), respectively. These numbers were higher in female and elderly groups. Patients experience a significant diagnostic delay of pSS and in the initiation of regular follow-up care. Targeted guardian programs or public health interventions are required to inform symptom interpretation and reduce delays.

## 1. Introduction

Primary Sjögren’s syndrome (pSS) is a chronic systemic autoimmune disease. The estimated annual incidence of pSS is 4–6 per 100,000 inhabitants for both sexes, according to population-based studies [1,2,3,4]. The hallmark of Sjögren’s syndrome is the lymphocytic infiltration of exocrine glandular tissues, which is characterized by dryness of the eyes [5] and mouth [6], resulting from impairment of the salivary and lachrymal gland function [7]. The diagnosis of pSS can be challenging because the cardinal features of sicca syndrome may be subclinical, may be attributed to other causes such as medication or the aging process, or may be misidentified as symptoms of other diseases.

pSS usually progresses slowly, with a benign course, slow deterioration in the salivary function, and no dramatic changes in symptoms. Many patients may remain undiagnosed as their presenting complaints are often subjective and non-specific. Numerous studies have documented an increased risk of non-Hodgkin’s lymphoma (NHL) [8,9,10,11,12,13,14] and other cancers [15,16,17,18] among pSS patients compared with the general population. Therefore, the early diagnosis of pSS is vital. Although prior studies have estimated the time of lag for diagnosis in patients with rheumatoid arthritis [19,20,21], little is known regarding the diagnostic delay of pSS.

This study aimed to assess the diagnostic delays in pSS. Using population-based universal health insurance claims data from Taiwan, we investigated the interval between the onset of sicca symptoms and a diagnosis of pSS.

## 2. Patients and Methods

### 2.1. Data Sources

The Taiwan National Health Insurance (NHI) program, which is a mandatory single-payer and universal coverage health insurance system, was implemented in 1995, with its coverage extending to 99.6% of Taiwan’s population by the end of 2011 [22]. The NHI research database containing the program’s claims data has been released to researchers in an electronically encrypted form since 1999 [23]. To avoid severe financial hardship for families coping with major injuries/illnesses, the NHI specifies 31 categories of catastrophic illness (e.g., cancers, hemophilia, autoimmune diseases, chronic renal failure, etc.) that are exempt from co-payment and/or co-insurance. The attending physician of a patient diagnosed as falling into one such category of catastrophic illness under the Ministry of Health and Welfare guidelines can submit related information in application for a catastrophic illness certificate (CIC). Applications are formally reviewed by a committee, and if approved, patients are then exempted from co-payment and/or co-insurance [24]. The CIC and ambulatory care expenditures by visit files of the NHI claims data were used in this study.

### 2.2. Patients and Study Design

This study is confined to patients approved for the CIC as a result of their pSS. To obtain a CIC for pSS, the patient’s attending physician is required to provide relevant clinical and laboratory information as part of the application for review, and the review committee assess applications according to the criteria of the American–European Consensus Group for pSS [25]. Excluded from the study were patients with multiple CICs, for pSS and additional autoimmune diseases, such as systemic lupus erythematosus (SLE), rheumatoid arthritis (RA), and other connective tissue diseases.

**Figure 1:**
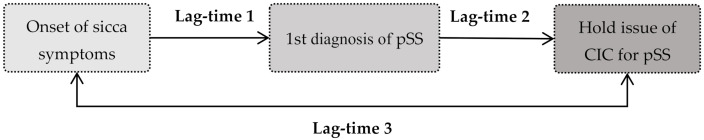
The time of lag before the diagnosis of primary Sjogren’s syndrome.

In designing this retrospective observational study, we included all patients with a first-time approved CIC for pSS (the International Classification of Diseases 9th revision Clinical Modification [ICD-9-CM] code 710.2) under NHI in 2011, and then sought to retrospectively identify the first documented symptoms/signs of dry eye and/or dry mouth via the ICD-9-CM 370.x (keratitis), 372.53 (conjunctival xerosis), 375.x (disorders of the lacrimal system), and 527.x (diseases of the salivary glands) standards. Records for all subjects were traced retrospectively, from the 2011 date of CIC approval by the NHI Administration to 1 January 2006. Three time period of lag were recorded, including between the initial recording of symptoms and the first diagnosis of pSS (lag-time 1), between the first diagnosis of pSS and issuance of a CIC for pSS (lag-time 2), and between the onset of symptoms and issuance of a CIC for pSS (lag-time 3) (Figure 1).

This study was conducted in accordance with the Declaration of Helsinki and the Declaration of Taipei on ethical considerations regarding Health Databases by the World Medical Association. It was approved by the Institutional Review Board of Kaohsiung Medical University Chung-Ho Memorial Hospital (IRB-NO: KMUH-IRB-EXEMPT-20140026).

### 2.3. Statistical Analysis

Descriptive statistics, including the median and interquartile range (IQR), were used to compare the lag-time weeks and the times of outpatient visits. The unit of delay time was weeks in this study. The Mann–Whitney U and Kruskal–Wallis tests were used to ascertain gender and age differences in the time of lag. All the statistical analyses were performed using SAS^®^ Enterprise Guide 7.0 (SAS Institute Inc., Cary, NC, USA) and IBM^®^ SPSS^®^ 21.0 (IBM Corp., New York, USA).

## 3. Results

**Figure 2:**
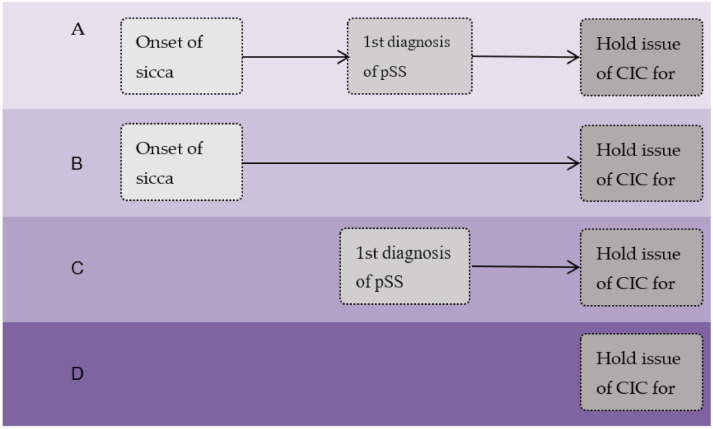
The patient groups of the diagnostic delay for primary Sjogren’s syndrome.

A total of 1970 patients with a CIC for pSS were included in the study. Four subgroups were identified according to the data recorded (Figure 2). Group A includes comprised of 900 patients (45.7%) with records showing the onset of pSS symptoms or signs (time point 1, T1), the first impression of pSS (time point 2, T2), and the date of CIC issuance for pSS (time point 3, T3) (T1→T2→T3). Group B is defined as 212 patients (10.8%) with a record of T1 and T3 (T1→T3), while group C represents 756 patients (38.4%) with records for T2 and T3 (T2→T3). Finally, group D represents 102 patients (5.2%) whose records only show a date of CIC issuance for pSS (T3 only) (Table 1).

**Figure 3:**
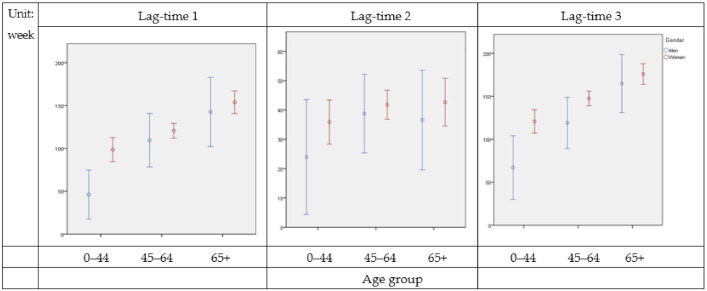
Lag time of sicca symptoms, diagnosis, and catastrophic illness certificate (CIC) issuance for primary Sjögren’s syndrome (pSS) by sex and age group.

The majority of patients were female (89%). The median time of lag between the first appearance of sicca symptoms and an initial diagnosis of pSS was 94 (IQR 12–182) weeks in men and 118 (IQR 30–208) weeks in women, with no age differentiation. The median time of lag from a diagnosis of pSS to the issuance of a CIC for the condition was 6 (IQR 2–37) weeks in men and 6 (IQR 2–37) weeks in women, also regardless of age. Additionally, the median for time between the first symptoms and CIC issuance for pSS was 113 (IQR 11–225) weeks in men and 155 (IQR 48–249) weeks in women (Table 2). Lag-time 1 and lag-time 3 exhibited statistical significance for different age groups and gender (Figure 3).

The median number of outpatient visits prior to the diagnosis of pSS was 2 (IQR 1–5) times in men and 3 (IQR 1–8) times in women. Female patients above 65 years of age required a median number of outpatient visits of 6 before the diagnosis of pSS (IQR 2–13). Further median calculations for outpatient visits are as follows: For visits between the first diagnosis of pSS and CIC issuance for the condition, 4 (IQR 2–6) times in men and 3 (IQR 2–7) times in women, and for visits taking place between initial signs of dry eyes and dry mouth, and issuance of a CIC specifically for pSS, 5 (IQR 2–12) times in men and 7 (IQR 3–15) times in women (Table 3).

## 4. Discussion

Primary Sjögren’s syndrome is a systemic autoimmune disease characterized by lacrimal and salivary gland dysfunction with resultant dryness of the eyes and mouth. The clinical presentation of dry eyes and dry mouth can be very subjective and may easily be ignored by patients with pSS. In our study, we examined the intervals occurring between the onset of pSS symptoms and diagnosis of the condition; the results suggest a significant delay from clinical symptoms to a definite diagnosis and the commencement of follow-up care for patients with pSS.

Our study observed that, for patients of all ages, the median time of lag before definite diagnosis was nearly 2 and a half years (113 weeks) in men and over 3 years (155 weeks) in women. A diagnosis of pSS was significantly more delayed for women than men, at 155 (IQR 48–249) weeks versus 113 (IQR 11–225) weeks. Elderly patients, both male and female, waited the longest for a definite diagnosis of pSS, with the most significant delays affecting women over 65 years of age, at 198 weeks (IQR 100–266). Patients in this group also needed the largest number of outpatient visits for a diagnosis of pSS to be confirmed.

In the period of delay between the initial signs of pSS and a definite pSS diagnosis, the median number of outpatient visits was 2 (IQR 1–5) times in men and 3 (IQR 1–8) times in women. For younger men (under 45 years of age), less time elapsed before a definite diagnosis was made, while for women in this age group, an initial impression of pSS was less delayed than for older women. Before the diagnosis of primary Sjogren’s syndrome, more outpatient visits were recorded for women than for men (7 vs. 5 times, respectively). The median time of lag between the first clinical acknowledgement of potential pSS and definite diagnosis of pSS (lag-time 2) was similar between groups and much shorter than the time of lag between the onset of symptoms and impression of pSS (lag-time 1), illustrating that once an impression of pSS has formed, the definitive diagnosis will follow without a significant delay.

Although some studies have reported a diagnostic delay issue in rheumatoid arthritis [20,21,26], psoriatic arthritis, and ankylosing spondylitis [19,27], to the best of our knowledge, this study is the first of its kind to use population-based data on primary Sjogren’s syndrome. First of all, the high validity of CIC-related diagnoses in the claims data has ensured that this data set provided a valuable opportunity to estimate the delay time for diagnosis in patients with pSS. Second, we had access to data on all of the outpatient clinics. Third, we ruled out all those who had secondary Sjogren’s syndrome, thus avoiding any over- or under-estimation of delay. A limitation of our study relates to the database, which did not record patient symptoms and signs, or laboratory data for pSS.

Patients with pSS are at a greater risk of developing not only NHL [8,9,10,11,12,13,14], but also other cancers [15,16,17,18], compared with the general population. In our previous study [18], most of these cancers were diagnosed during the first 2 years following the diagnosis of pSS. Early diagnosis and the initiation of regular follow-up can prevent the delayed detection of cancers in patients with pSS. A person’s educational level, cultural attitudes, and a lack of awareness regarding the disease might influence lag times for the diagnosis of pSS. The further provision and application of educational material to increase the awareness of doctors and patients is very important.

## 5. Conclusions

Our study highlights the significant diagnostic delay of primary Sjögren’s syndrome and in the initiation of regular follow-up care. Targeted public health interventions are required to inform symptom interpretation and reduce delays.

## Figures and Tables

**Table 1:** Number of patients in different groups and gender.

Group	Total		Female		Male	
	*n*	(%)	*n*	(%)	*n*	(%)
A	900	45.7	820	46.6	80	37.7
B	212	10.8	186	10.6	26	12.3
C	756	38.4	667	37.9	89	42.0
D	102	5.2	85	4.8	17	8.0

**Table 2:** Three items of the diagnostic delay in primary Sjögren’s syndrome (unit: weeks).

Gender Age Group	Lag-Time 1		Lag-Time 2		Lag-Time 3	
	Median	IQR	Median	IQR	Median	IQR
Female						
All ages	118	30–208	6	2–37	155	48–249
0–44	72	19–177	5	2–29	105	31–212
45–64	110	22–196	6	2–41	152	37–249
≥65	160	70–244	5	2–38	198	100–266
Male						
All ages	94	12–182	6	2–37	113	11–225
0–44	15	3–89	4	1–11	27	6–113
45–64	102	12–184	7	3–48	102	6–213
≥65	136	32–254	6	3–37	188	89–259

IQR, interquartile range.

**Table 3:** Number of outpatient visits of three lag times for primary Sjögren’s syndrome.G

Gender Age Group	Lag-Time 1		Lag-Time 2		Lag-Time 3	
	Median	IQR	Median	IQR	Median	IQR
Female						
All ages	3	1–8	3	2–7	7	3–15
0–44	2	1–4	3	2–6	5	2–10
45–64	3	1–7	4	2–7	7	4–14
≥65	6	2–13	4	2–8	9	4–20
Male						
All ages	2	1–5	4	2–6	5	2–12
0–44	1	1–1	3	1–4	4	2–6
45–64	4	1–7	4	2–7	5	2–14
≥65	2	1–5	4	2–7	6	4–13

IQR, interquartile range.

## Data Availability

Data are available from the National Health Insurance (NHI) research database published by the Taiwan NHI administration. Due to the legal restrictions imposed by the government of Taiwan concerning the Personal Information Protection Act, the data cannot be made publicly available.

## References

[1] Pillemer S.R., Matteson E.L., Jacobsson L.T., Martens P.B., Melton L.J., O’Fallon W.M., Fox P.C. (2001). Incidence of physician-diagnosed primary Sjögren syndrome in residents of Olmsted County, Minnesota. Mayo Clin. Proc..

[2] Plesivcnik Novljan M., Rozman B., Hocevar A., Grmek M., Kveder T., Tomsic M. (2004). Incidence of primary Sjogren’s syndrome in Slovenia. Ann. Rheum. Dis..

[3] Alamanos Y., Tsifetaki N., Voulgari P.V., Venetsanopoulou A.I., Siozos C., Drosos A.A. (2006). Epidemiology of primary Sjögren’s syndrome in north-west Greece, 1982–2003. Rheumatology..

[4] Weng M.Y., Huang Y.T., Liu M.F., Lu T.H. (2011). Incidence and mortality of treated primary Sjogren’s syndrome in Taiwan: A population-based study. J. Rheumatol..

[5] Jacobsson L.T., Axell T.E., Hansen B.U., Henricsson V.J., Larsson A., Lieberkind K., Lilja B., Manthorpe R. (1989). Dry eyes or mouth--an epidemiological study in Swedish adults, with special reference to primary Sjögren’s syndrome. J. Autoimmun..

[6] Vitali C., Bombardieri S., Moutsopoulos H.M., Balestrieri G., Bencivelli W., Bernstein R.M., Bjerrum K.B., Braga S., Coll J., de Vita S. (1993). Preliminary criteria for the classification of Sjögren’s syndrome. Results of a prospective concerted action supported by the European Community. Arthritis Rheum..

[7] Ramos-Casals M., Tzioufas A.G., Font J. (2005). Primary Sjögren’s syndrome: New clinical and therapeutic concepts. Ann. Rheum. Dis..

[8] Kassan S.S., Thomas T.L., Moutsopoulos H.M., Hoover R., Kimberly R.P., Budman D.R., Costa J., Decker J.L., Chused T.M. (1978). Increased risk of lymphoma in sicca syndrome. Ann. Intern. Med..

[9] Kauppi M., Pukkala E., Isomäki H. (1997). Elevated incidence of hematologic malignancies in patients with Sjögren’s syndrome compared with patients with rheumatoid arthritis (Finland). Cancer Causes Control..

[10] Valesini G., Priori R., Bavoillot D., Osborn J., Danieli M.G., Del Papa N., Gerli R., Pietrogrande M., Sabbadini M.G., Silvestris F. (1997). Differential risk of non-Hodgkin’s lymphoma in Italian patients with primary Sjögren’s syndrome. J. Rheumatol..

[11] Davidson B.K., Kelly C.A., Griffiths I.D. (1999). Primary Sjögren’s syndrome in the North East of England: A long-term follow-up study. Rheumatology.

[12] Voulgarelis M., Dafni U.G., Isenberg D.A., Moutsopoulos H.M. (1999). Malignant lymphoma in primary Sjögren’s syndrome: A multicenter, retrospective, clinical study by the European Concerted Action on Sjögren’s Syndrome. Arthritis Rheum..

[13] Pertovaara M., Pukkala E., Laippala P., Miettinen A., Pasternack A. (2001). A longitudinal cohort study of Finnish patients with primary Sjögren’s syndrome: Clinical, immunological, and epidemiological aspects. Ann. Rheum. Dis..

[14] Anderson L.A., Gadalla S., Morton L.M., Landgren O., Pfeiffer R., Warren J.L., Berndt S.I., Ricker W., Parsons R., Engels E.A. (2009). Population-based study of autoimmune conditions and the risk of specific lymphoid malignancies. Int. J. Cancer..

[15] Lazarus M.N., Robinson D., Mak V., Møller H., Isenberg D.A. (2006). Incidence of cancer in a cohort of patients with primary Sjogren’s syndrome. Rheumatology.

[16] Zhang W., Feng S., Yan S., Zhao Y., Li M., Sun J., Zhang F.C., Cui Q., Dong Y. (2010). Incidence of malignancy in primary Sjogren’s syndrome in a Chinese cohort. Rheumatology.

[17] Theander E., Henriksson G., Ljungberg O., Mandl T., Manthorpe R., Jacobsson L.T. (2006). Lymphoma and other malignancies in primary Sjögren’s syndrome: A cohort study on cancer incidence and lymphoma predictors. Ann. Rheum. Dis..

[18] Weng M.Y., Huang Y.T., Liu M.F., Lu T.H. (2012). Incidence of cancer in a nationwide population cohort of 7852 patients with primary Sjogren’s syndrome in Taiwan. Ann. Rheum. Dis..

[19] Bashda H., Kong K.O., Tak P.P. (2007). Rheumatoid arthritis in Dubai: Delayed diagnosis and low usage of disease modifying antirheumatic drugs. Ann. Rheum. Dis..

[20] Chan K.W., Nelson D.T., Walter A.M. (1994). The lag time between onset of symptoms and diagnosis of rheumatoid arthritis. Arthritis Rheum..

[21] Rogríguez-Polanco E., Al Snih S., Kuo Y.F., Millán A., Rodríguez M.A. (2011). Lag time between onset of symptoms and diagnosis in Venezuelan patients with rheumatoid arthritis. Rheumatol. Int..

[22] Department of Health, Taiwan (2012). 2012 Taiwan Public Health Report.

[23] Hsihe C.Y., Su C.C., Shao S.C., Sung S.F., Lin S.J., Kao Y.H., Lai E.C.C. (2019). Taiwan’s National Health Insurance Research Database: Past and future. Clin. Epidemiol..

[24] Bureau of National Health Insurance Regulations for Exempting NHI Insured Persons from the Co-Payment. https://www.nhi.gov.tw/english/Content_List.aspx?n=E5509C8FE29950EA&topn=1D1ECC54F86E9050.

[25] Vitali C., Bombardieri S., Jonsson R., Moutsopoulos H.M., Alexander E.L., Carsons S.E., Daniels T.E., Fox P.C., Fox R.I., Kassan S.S. (2002). Classification criteria for Sjögren’s syndrome: A revised version of the European criteria proposed by the American-European Consensus Group. Ann. Rheum. Dis..

[26] Stack R.J., Simons G., Kumar K., Mallen C.D., Raza K. (2013). Patient delays in seeking help at the onset of rheumatoid arthritis: The problem, its causes and potential solutions. Aging Health.

[27] Sørensen J., Hetland M.L. (2015). All departments of rheumatology in Denmark. Diagnostic delay in patients with rheumatoid arthritis, psoriatic arthritis and ankylosing spondylitis: Results from the Danish nationwide DANBIO registry. Ann. Rheum Dis..

